# Design and performance test of variable diameter threshing drum of combine harvester

**DOI:** 10.1002/fsn3.2402

**Published:** 2021-06-30

**Authors:** Zhan Su, Zhao Ding, Liquan Tian, Xue Lin, Zhiming Wang

**Affiliations:** ^1^ Key Laboratory of Crop Harvesting Equipment Technology of Zhejiang Province College of Engineering Jinhua Polytechnic Jinhua China

**Keywords:** combine harvester, field test, rice, threshing gap, variable diameter threshing drum

## Abstract

During the rice harvesting process of combine harvester, it is necessary to adjust the working parameters of the threshing device in time according to working conditions to ensure operational efficiency and performance. This paper designs a variable diameter threshing drum, develops a device for diameter regulation with self‐locking function, and its installation process and working principle are introduced in detail. A force analysis and modal analysis were carried out on the device for diameter adjustment of variable diameter threshing drum. It aims to ensure that the design dimensions of the constant speed spiral disc and claws in the device for diameter adjustment are in compliance with the self‐locking conditions, and effectively ensure the reliability of the device for diameter adjustment. A field experiment was carried out to check the reliability of the developed system. A regression analysis model takes the feeding rate, threshing gap and drum speed as influencing factors. The grain separation loss rate and damage rate as performance indexes were established, and calculations for multiple target optimization were performed. The experimental results showed that the threshing gap could be adjusted in real time according to the different feeding rates of the variable diameter threshing drum.

## Graphical abstract

This paper develops a threshing drum with variable diameter making it possible to adjust the drum diameter integrally and quickly. The developed device can provide a reference to design of threshing drum with variable diameter as well as that of self‐adaptive control system.

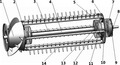

**Highlights**

This research was aimed at assessing the performance of variable diameter threshing drum.
The variable diameter threshing drum can change the drum quickly and directly.
The variable diameter threshing drum can effectively reduce the harvest loss.

## 1 INTRODUCTION

Threshing device is one of the important parts of combine harvesters, whose function determines the working performance, efficiency, and adaptability of the whole machine directly. Threshing gap refers to the clearance between the threshing elements of the drum and the concave grid. During field harvesting, the actual feeding rate shows a great fluctuation due to various biomechanical characteristics and growth density of different varieties of rice, which will deteriorate the threshing and separation performance of combine harvester (Toshikazu and Tatsuro, 2017; Alizadeh and Bagheri, 2009; Singh and Singh, 1981). Therefore, it is necessary to adjust the threshing gap in time according to various working conditions to ensure the operation stability of combine harvesters and improve its harvesting adaptability.

Researchers have conducted a lot of studies on the effect of threshing gap on threshing performance, as well as its regulation mode. Scholars have established some basic models of threshing and separation process through comprehensive consideration of multiple factors that exist during the threshing process. They optimized and improved the model in terms of threshing drum rotational speed, threshing gap, material flow rate and feeding rate, the law of influence exerted by various factor on the index of performance of threshing has been made clear (Osueke, 2011; Tang et al., 2011; Valentin et al., 2009; Hosoi et al., 2008). At the same time, on the basis of the irregular motion of material in the nonuniform helical path between the threshing drum and the concave screen, the kinematics mathematical model for materials flow in axial flow threshing and separating device and the grain separating loss prediction model were established which provides a design basis for designing and adjusting the working parameters of threshing devices (Miu and Kutzbach, 2008).

There exist two ways to adjust the threshing gap:

One is by means of changing the position of the concave grid. For example, hinging two ends of the concave grid with four mounting seats, placing the middle part of the concave grid on the cross beam. Adjusting the cross beam by moving the connecting rod mechanism downwards or upwards, resulting in the consequent fall or rise of the middle part of the concave grid and increase or decrease in threshing gap. Hinge one end of the concave grid with the rack, and hinge the other end with the piston rod of the hydraulic cylinder, and achieve the adjustment of the clearance of the concave grid by means of expansion of the piston rod of the hydraulic cylinder (Matousek et al., 2017; Bergkamp, 2015; Regier and Robert, 2012). However, this method of threshing gap adjustment by means of changing the relative position of the concave screen. It only works for the threshing gap between the bottom of the drum and the concave grid. Having effect on neither that on both sides of the drum nor that between the top of the drum and the top guide plate of the top cover. It leads to the unequal threshing gap and poor fluidity of materials in the threshing device, and its deteriorated delivery capacity and operation performance (Su et al., 2020; Tang et al., 2013).

The other approach is to adjust the threshing gap by increasing or decreasing the diameter of the drum. Although this method can ensure the uniformity of the threshing gap in the threshing device along the circumferential direction of the drum, in most of the existing threshing drums, the threshing tooth rod is fixed on several supporting plates through bolts. On one hand, when adjusting the drum diameter, it generally requires changing one by one the location of the lower connection seat of each threshing tooth rod relative to the supporting plate of each threshing drum after the combine harvester stops working, and on the other hand, the adjustable drum diameter series is limited (Tang et al., 2014; Xu and Li, 2014; GIvan et al., 2014), it is not suitable for real‐time adjustment in the field operation.

Based on the existing threshing drum of combine harvesters, by adding a device for diameter adjustment and a self‐locking device. This paper develops a threshing drum with variable diameter making it possible to adjust the drum diameter integrally and quickly, which threshing performance is verified through a field experiment. The developed device can provide a reference to the design of threshing drum with a variable diameter as well as that of a self‐adaptive control system.

## 2 MATERIALS AND METHOD

### 2.1 Design of feeding section

**FIGURE 1:**
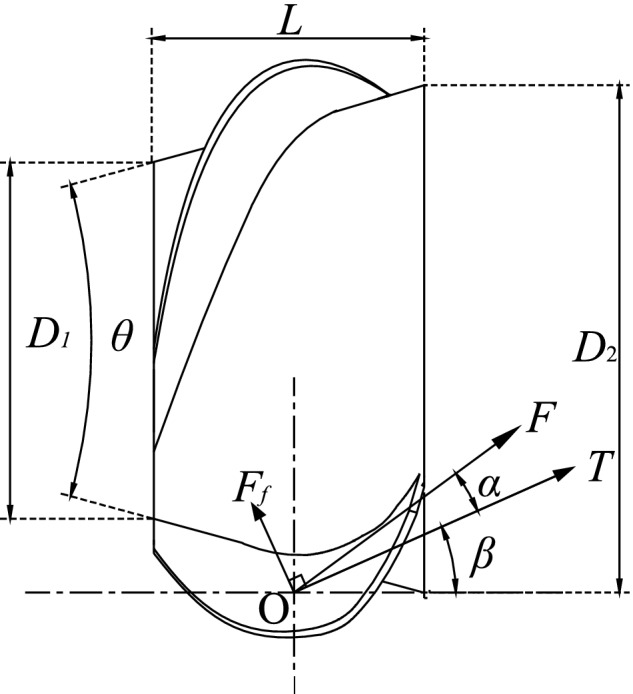
Schematic diagram of material force analysis

The feeder‐beater smoothly feeds the material conveyed by the transportation device into the threshing drum for threshing. In order to ensure a smooth feeding and avoid material blockage, the material conveyed at any point O of the feeder‐beater undergoes a force analysis, as shown in Figure 1.

As seen from Figure 1, there are frictional force, Ff between helical blade and material; normal thrust, T that the frictional force exerts on the material; the resultant force, F and the normal thrust, T deviate from an angle α, which is the friction angle between material and helical blade. In order to ensure the smooth completion of the axial materials conveying, it is necessary to meet the condition that the axial conveying force is greater than the axial direction resistance (Qu et al., 2018), that is,

(1) $$Tcos\beta >F_{f}sin\beta$$

(2) $$F_{f}=Ttan\alpha$$

where, *β* is the helical angle of the helical blade; *α* is the friction angle between the material; the helical blade is 17°

From formula (1) and formula (2), the conditions for the smooth transportation of materials along the feeder‐beater are as follows: *β* < 90°–*α* = 73°, the helical angle of the helical blade is selected to be 30°. Feeder‐beater wheel length:

(3) $$L=\frac{S}{K}$$

The feeder‐beater designed in this paper is a frustum structure with a cone angle *θ* = 17° and a helical lead of *S* = 700 and the number of helical heads *K* = 2. As obtained from formula (3), the length of the feeder‐beater *L* = 350 mm, the diameter of the front end of feeder‐beater *D_1_* = 300 mm, and the diameter of the rear end of feeder‐beater *D_2_* = 410 mm.

### 2.2 Design of threshing and separating section

The length of the threshing part of the drum determines its threshing and separating ability. The drum should be long enough so that the material can be fully separated by threshing. Limited by the length of threshing drum of the combine harvester, the installation position of the device for self‐locking adjustment should be reserved on the premise that material can be fully threshed and separated. Therefore, the length of threshing and separation section of drum were recalculated. The formula for calculating the length of drum threshing section is as follows:

(4) $$L=a\left(\frac{Z}{K}‐1\right)+2\Delta l$$

where, *a* is the distance between the teeth trace, generally 25–50 mm, takes 50 mm; *Z* is the total number of tooth 81; *K* is the number of screw heads; that is, the number of spike tooth on each tooth trace is 3; *ΔL* is the distance between the end rod teeth and the end of the spoke, and take 75 mm for the threshing device.

Formula (4) shows that the length of the threshing and separation section of the threshing drum is *L* = 1,450 mm, the length of the feeding section *S* = 350 mm, and the total length of the original threshing drum is 2,100 mm, so the reserved length of the self‐locking part is *D* = 300 mm.

### 2.3 Design and force analysis of device for diameter adjustment

**FIGURE 2:**
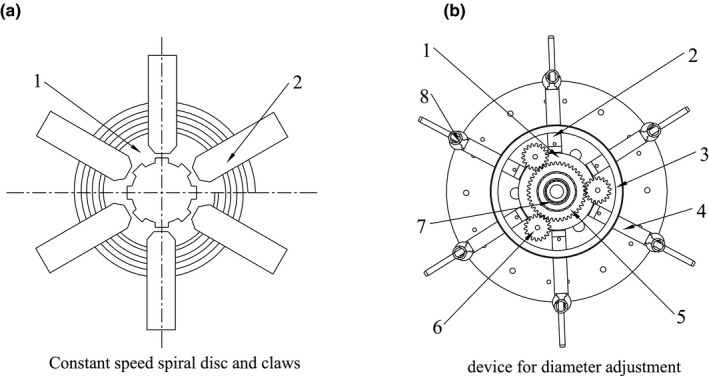
Structure diagram of device for diameter adjustment. 1. Constant speed spiral disc 2. Claw 3. Limit disc 4. Connecting rod 5. Sun wheel 6. Planet wheel 7. Spline sleeve 8. Threshing tooth rod, (a) Constant speed spiral disc and claws, (b) device for diameter adjustment

As the core parts of the diameter adjustment device, constant speed screw disc and matching claws were designed according to the Archimedes spiral principle. As shown in Figure 2a, the inner end face of the constant speed spiral plate is equipped with plane threads, the driving thread pitch of the threads is *T* = 8 mm, the spiral diameter is *D* = 150 mm. The thread teeth are embedded in one end plane of the six claws. The overall structure of the device is shown in Figure 2b, which consists of a constant speed spiral plate, limit disc, claws, connecting rod, sun wheel, planet wheel, and spline sleeve. The constant speed spiral disc and sun wheel are jointly installed on the spline sleeve, the spline hub is installed on the roller shaft, and the limit disc is fixed on the support plates, where six claws are connected through each of the six I‐shaped grooves, respectively. One end connected with the claw engages with the plane thread in the constant speed spiral disc, the other end is welded with the connecting rod, and the connecting rod is welded with the threshing tooth rod.

In order to ensure the operation stability of variable diameter threshing drum and prevent the radial displacement of claw caused by the force of threshing tooth rod and avoid the consequent rotation of constant speed spiral disc, force analysis was carried out for the constant speed spiral disc and claw in the device for diameter regulation.

**FIGURE 3:**
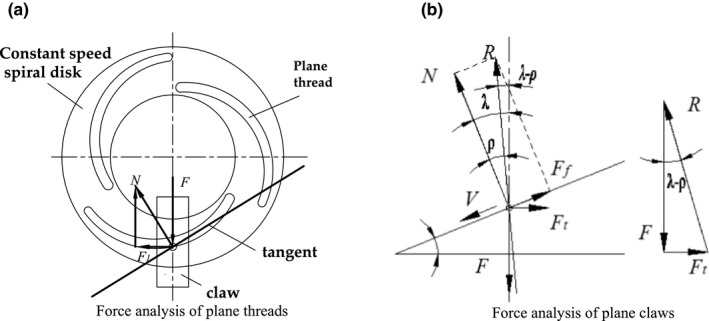
Force analysis of plane threads and claws, (a) Force analysis of plane threads, (b) Force analysis of plane claws

The plane threads on the constant speed spiral disc are simplified to single‐tooth plane threads for force analysis. The claw is taken as a research object. As shown in Figure 3a, it is assumed that the centrifugal force *F* acting on the claw is concentrated on the meshing points on the spiral, the counter force N is perpendicular to the tangent of the meshing points, and the inclination angle of the tangent line *λ* is the helix one. For further force analysis of meshing point, as shown in Figure , when the claw moves downwards under centrifugal force, the friction *F_f_* goes upwards. In the figure, *R* is the resultant force of normal reaction *N* and friction force *F_f_*, *F_t_* is the horizontal force component of *R*, friction angle *ρ* is the angle between *R* and *N*, and the angle between *R* and centrifugal force *F* is (*λ–ρ*).

(5) $$F_{t}=Ftan\left(\lambda ‐\rho \right)$$

In the formula, *F_t_* is the circumferential force driving the rotation of the constant speed spiral disc; *F* is the radial force driving the movement of the connecting claw; thread lead angle λ = arctan (T/πD), *T*: screw pitch of thread transmission, *D*: thread diameter; *ρ* is the friction angle of thread transmission, *ρ* = *arctan f*, and *f* is the friction coefficient.

Formula (5) shows that if *λ* < *ρ*, then *F_t_* is negative. As long as the condition *λ* < *ρ* *is* met, regardless of the magnitude of the centrifugal force *F*, the claw will not move radially under its action, and self‐locking can be formed. The lowest friction coefficient of steel is *f* = 0.05, and the lowest friction angle *ρ* is 3°.

(6) $$\lambda =\arctan\left(\frac{T}{\pi D}\right)<\rho =arctanf$$

(7) $$\left(\frac{T}{\pi D}\right)<f=0.05$$

(8) $$\frac{T}{D}<0.05\pi =0.16$$

The plane threads of constant speed spiral disc adopt pitch *T* = 8, diameter *D* = 150, *T/D* = *8/150* = 0.053, in full compliance with the self‐locking condition of *T/D* < 0.16.

### 2.4 Device for self‐locking adjustment

**FIGURE 4:**
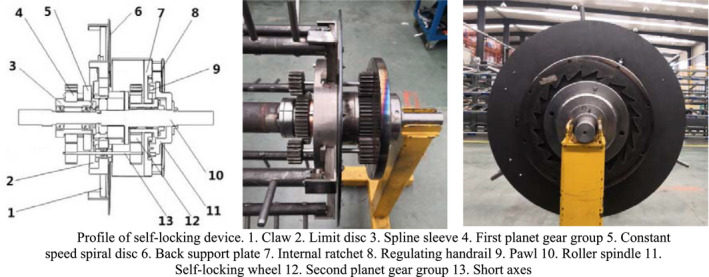
Profile of self‐locking device. 1. Claw 2. Limit disc 3. Spline sleeve 4. First planet gear group 5. Constant speed spiral disc 6. Back support plate 7. Internal ratchet 8. Regulating handrail 9. Pawl 10. Roller spindle 11. Self‐locking wheel 12. Second planet gear group 13. Short axes

Through the force analysis for diameter adjustment, under the premise that the effective self‐lock of the drum can be ensured in the process of rotation. The device for self‐locking adjustment is installed at the tail of the variable diameter threshing drum to provide power input and ensure its stability. As shown in Figure 4, it is divided into two parts: transmission mechanism and self‐locking mechanism.

The transmission mechanism is composed of the first planet gear group installed on the inner side of the rear support plate and the second one installed on the outer side. The sun wheel in the first planet gear group is connected with the constant speed spiral disc through the spline sleeve, and gear group in the second planet is connected with the inner ratchet in the self‐locking device through the spline sleeve. The planetary wheels in the center are connected by three short axes which run through the supporting plate. The self‐locking mechanism includes inner ratchet, pawl, and self‐locking wheel. The inner ratchet and the sun wheel in the second planet gear group are jointly installed on the spline sleeve. The outer side of the inner ratchet is equipped with adjusting handrails. The self‐locking wheel is fixed on the roller shaft through bolts. A pair of pawls is set on the end face of the self‐locking wheel. Spring plates are set on one side of the pawl to ensure the engagement of the pawl and the inner ratchet. The direction of ratchet teeth of an internal ratchet is opposite to the rotation direction of the drum, which can ensure that the self‐locking wheel fixed on the drum shaft can lock the internal ratchet wheel through the pawl during the rotation process of the drum to guarantee no rotation happening to the transmission device under the action of external forces.

### 2.5 Rice varieties and farming area test methods

In this experiment, a self‐developed combine harvester was used, in which an online monitoring device was installed for monitoring grain impurity content, damage rate, loss rate, drum rotation speed, and torque sensor with control functions of adjusting the header height, drum rotation speed, chaffer opening, and forward speed, making it possible to real‐time monitor the operation parameters and performance parameters of the combine harvester (Chen et al., 2018, 2019; Liang et al., 2016). The experiment field was located in Wujiang, Suzhou, Jiangsu Province. The tested rice was Jia Hua, and some of its characteristics are shown in Table 1.

**TABLE 1:** Characteristics of Rice Material

Parameter	Numerical value
Crop height (cm)	79.2
Crop density (plants/m^2^)	110
Plant spacing (cm)	14
Spike head height (cm)	16.1
Grain moisture content (%)	20.6
Straw moisture content (%)	70.8
MOG (material other than grain)/Grain ratio	2.85
Number of grains per spike seed	135
Weight of 1,000 grains (g)	32.4

Before the test, several fields with uniform rice growth were selected and tested. The harvesting length was 20 m in length for each test. The forward speed of the combine harvester was maintained within 1.0–1.2 m/s, to ensure the feeding rate. In the field test, the average cutting width is 2.58 m, the stubble height is 15 cm. After the material is separated by the threshing drum, the stalk is discharged from the grass discharge port and falls on the tarpaulin behind the combine harvester. After the test, the quality of stalks on the tarpaulin, the grain contained in the straw, and the unpurified grain were collected manually to obtain the quantity of stalk, the rate of separation loss and the grain damage rate. The data obtained artificially were compared with those collected by the combine harvester by means of online monitoring to get the final degranulation performance data (Chen et al., 2020). Design‐Expert software (Stat‐Ease) is used for regression analysis of test data, and the nonsignificant items are eliminated at the significant level when α = 0.05 and the obtained values for simplified regression equations of separation loss rate *Y_1_* and damage rate *Y_2_*.

### 2.6 Variable diameter drum modality analysis

The simplified variable diameter drum model was introduced into ANSYS Workbench, then set the attribute of the material of Q235A for the variable diameter drum and 45# steel of the drum center shaft, as shown in Table 2. After that, grid division was implemented on the variable diameter drum; to minimize the calculation scale and obtain better effect, intelligent‐free element division was adopted, and part of the parameters was defined at the time of grid division considering the complex structural shape of the variable diameter drum, and the impact of calculation accuracy and calculation time. The element size was set as 5 mm, and the remaining parameters are set as default. At last, zero friction constraints were applied at both ends of the main shaft of the variable diameter drum, and the rotatable load was applied at one end, whose rotation speed was defined as 600 rad/min.

**TABLE 2:** Q235A and 45# material properties

Material	Density/(kg·m^−3^)	Poisson's ratio	Elastic modulus	Yield strength/Mpa	Tensile strength/Mpa
Q235A	7.86*10^3^	0.288	2.12*10^11^	235	390
45#	7.89*10^3^	0.269	2.09*10^11^	355	600

The software of ANSYS is utilized in the modal analysis of the variable diameter threshing drum. In most cases, low‐order frequency shows a higher impact on the structure and a stronger destructive effect. To have a comprehensive idea of the threshing drum, analysis is carried out on the inherent frequency of the first six orders of the threshing drum considering the fact that any object is furnished with the translation in three directions and rotation in three directions, that is, six degrees of freedom in total.

### 2.7 Dynamic balance detection of variable diameter drum

**FIGURE 5:**
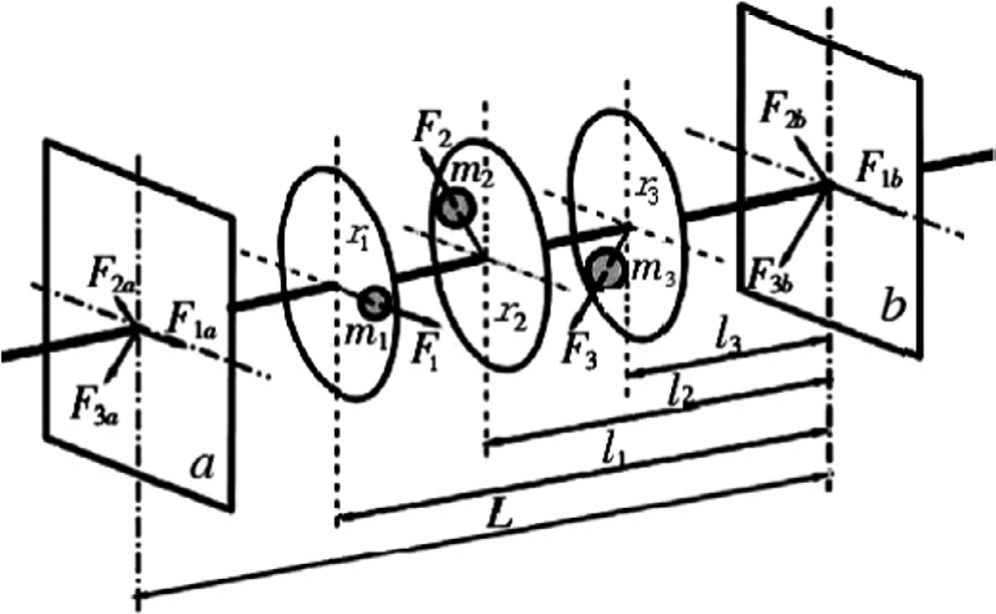
Dynamic balance schematic diagram

Dynamic balance simulation can obtain the variation law of bearing reaction force, resultant force, and dynamically unbalanced residual value of both ends of threshing drum (Meusel et al., 2018). The dynamic unbalanced residual value can be removed by means of theoretical calculation to achieve a dynamically balanced state of threshing drum (Zhang and Zhou, 2008). The principle of dynamic balance is shown in Figure 5, different eccentric mass exists in three planes, that is, m_1_, m_2_, and m_3_, and the corresponding inertial centrifugal force F_i_ (i = 1, 2, and 3) can be decomposed into the resultant force of F_ia_ and F_ib_ in the balance basis surface of a and b with the balance relationship as:

(9) $$F_{\mathrm{ia}}=\frac{F_{i}l_{i}}{L},F_{\mathrm{ib}}=\frac{F_{i}\left(L‐l_{i}\right)}{L}\left(i=1,2,3\right)$$

Then, the balance mass was adjusted in Plane a and Plane b until both the resultant force of inertia and the resultant couple of the inertia couple reach 0, that is, ΣF=0 and ΣM=0, thereby realizing the dynamic balance of the drum.

**FIGURE 6:**
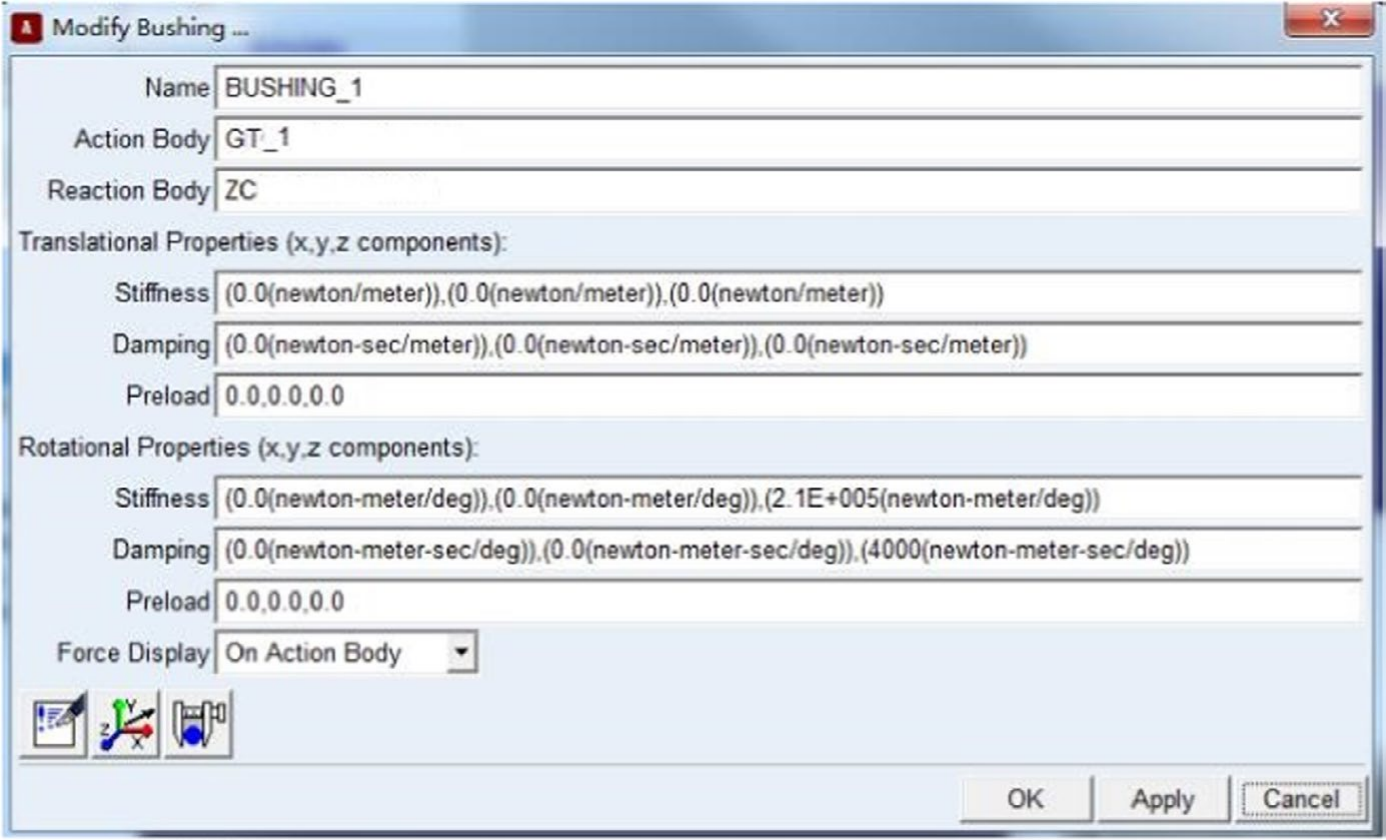
The Parameters of bushing

**FIGURE 7:**
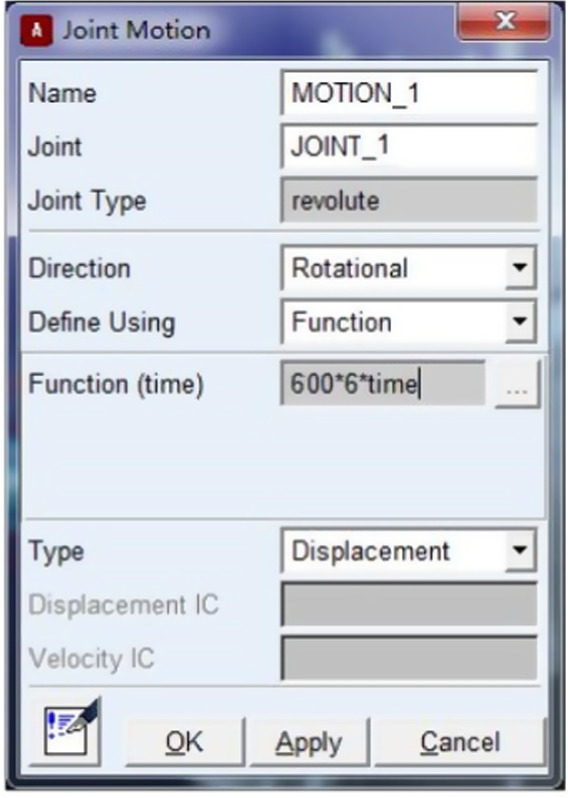
The Parameters setting of joint motion

Set the parameters necessary to establish the simulation model of drum dynamic balance in ADAMS environment, the driving device, coupling, the supporter at End A and that at End B share the same axis, and the supporter at End A and that at End B are supported by the spherical joint (i.e., the rotor is connected with the ground). The coupling was simulated with bushing of which the parameters are shown in Figure 6. A part was built in the drive to establish the rotating joint with the ground, and the rotation axis shares the same axis with the supporter at End A and that at End B of the rotor, thus bringing motion to the rotating coupling, the motion parameters are shown in Figure 7. Input 600n*6.0*time (n refers to the rotation speed, r/min.) into the column of “Function,” and the impact of the gravity on the calculation result was not considered in the simulation model. To ensure the solution precision, the residual error is ≤0.001 in the setting of solution parameters.

## 3 RESULTS AND DISCUSSION

### 3.1 Assembly of variable diameter threshing drum

**FIGURE 8:**
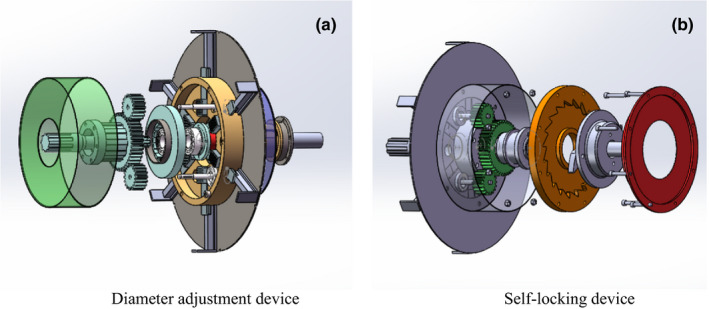
Installation flowchart of feeder‐beater and self‐locking device. (a) Diameter adjustment device, (b) Self‐locking device

Referring to Figure 8a for the assembly of the diameter adjustment device, first, the limit disc was fixed onto the supporting plate, making sure their axis was aligned with that of the main shaft. Second, the six claws and the connecting rod passed through the limit disc, and clung to the supporting plate, after that, the installation spline of the constant speed spiral disc was sheathed before being installed inside the limit disc. When the engagement between the end surface thread of the constant speed spiral disc and the groove of the claw was achieved, the sun wheel in the first planet gear group was installed on the spline sleeve. Then, the three planet gears were installed on the connecting rod. At last, the dust cover was installed.

Referring to Figure 8b for the assembly of the adjustment and self‐locking device, first, the sun wheel of the second planet gear group was installed outside the supporting plate. After that, three planet gears were installed on the connecting rod, respectively, which was engaged with the sun wheel installed in the center. Thus, the linkage between the first and the second planet gear groups was installed on both sides of the rear supporting plate. Second, the inner ratchet was installed on the flat key sleeve, and the bearing exists at the connection between the inside of the flat key sleeve and the main shaft of the roller. After that, the claws of the self‐locking wheel and the ratchet of the inner ratchet wheel were engaged, and the inner ratchet wheel was fixed onto the roller shaft. At last, the adjustment plate was fixed with the inner ratchet wheel by bolts, in this manner, adjustment is available through the rotation of the inner ratchet wheel.

**FIGURE 9:**
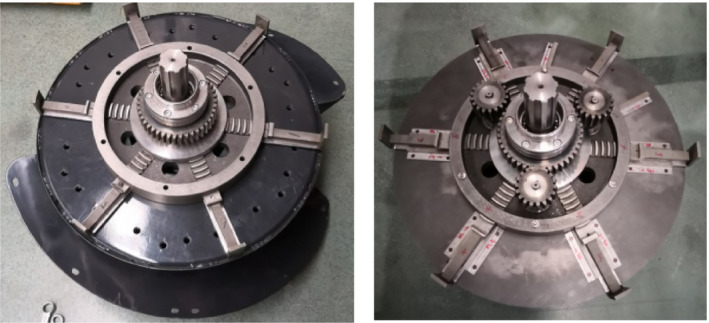
Physical drawing of feeder‐beater and self‐locking device

A set of devices for diameter adjustment was installed on the supporting plate behind the feeder‐beater. Another set of diameter adjusting devices was installed on the rear supporting plate, as shown in Figure 9.

**FIGURE 10:**
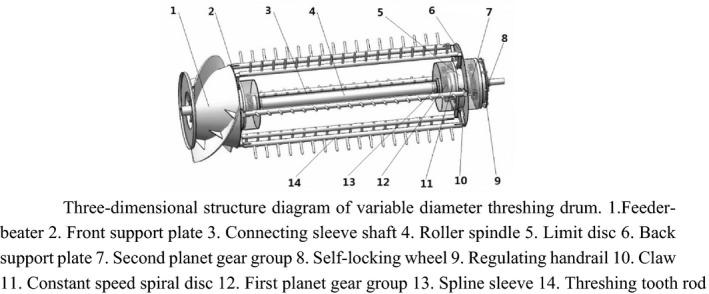
Three‐dimensional structure diagram of variable diameter threshing drum. 1. Feeder‐beater 2. Front support plate 3. Connecting sleeve shaft 4. Roller spindle 5. Limit disc 6. Back support plate 7. Second planet gear group 8. Self‐locking wheel 9. Regulating handrail 10. Claw 11. Constant speed spiral disc 12. First planet gear group 13. Spline sleeve 14. Threshing tooth rod

The section of feeder‐beater and the device for self‐locking adjustment are installed at both ends of the threshing separation part to form a complete variable diameter threshing drum. The feeder‐beater and the device for self‐locking adjustment are connected by the spindle and six threshing tooth rods. The two groups of devices of diameter adjustment are connected by the connecting sleeves, the complete variable diameter threshing drum as shown in Figure 10.

The working principle:

When the drum needs to adjust its diameter, the bolts fixing the self‐locking wheel with the drum shaft are unscrewed at first, and the self‐locking wheel can rotate with the inner ratchet wheel; the inner ratchet wheel can rotate by adjusting the handrail, and the internal ratchet rotates the sun and planet wheels in the second planet gear group through sheaves. The rotation of the sun wheel in the first planet gear group results in rotation of the constant speed spiral disc in the device for diameter adjustment. The rotation of the plane threads on the constant speed spiral disc leads to the radial reciprocating motion of the claw along the I‐shaped groove of the limit plate, changing the relative position of the threshing tooth rod at the top of the claw. At the same time, the two groups of devices for diameter adjustment are linked by connecting sleeve shaft, which can realize the whole and fast adjustments of the diameter of threshing drum. Through the transmission mechanism, it can be known that every turn of the internal ratchet is equivalent to one turn of the constant speed spiral disc. The displacement of the claw, S is equal to the thread pitch, T of the plane thread (i.e., *S* = *T* = 8 mm) and the tooth number of the internal ratchet *Z* = 20. Therefore, the claw displaces *L* = *S*/*Z* = 0.4 mm for each turn of the internal ratchet. Therefore, the diameter adjustment of the drum can reach 0.4 mm per stage.

**FIGURE 11:**
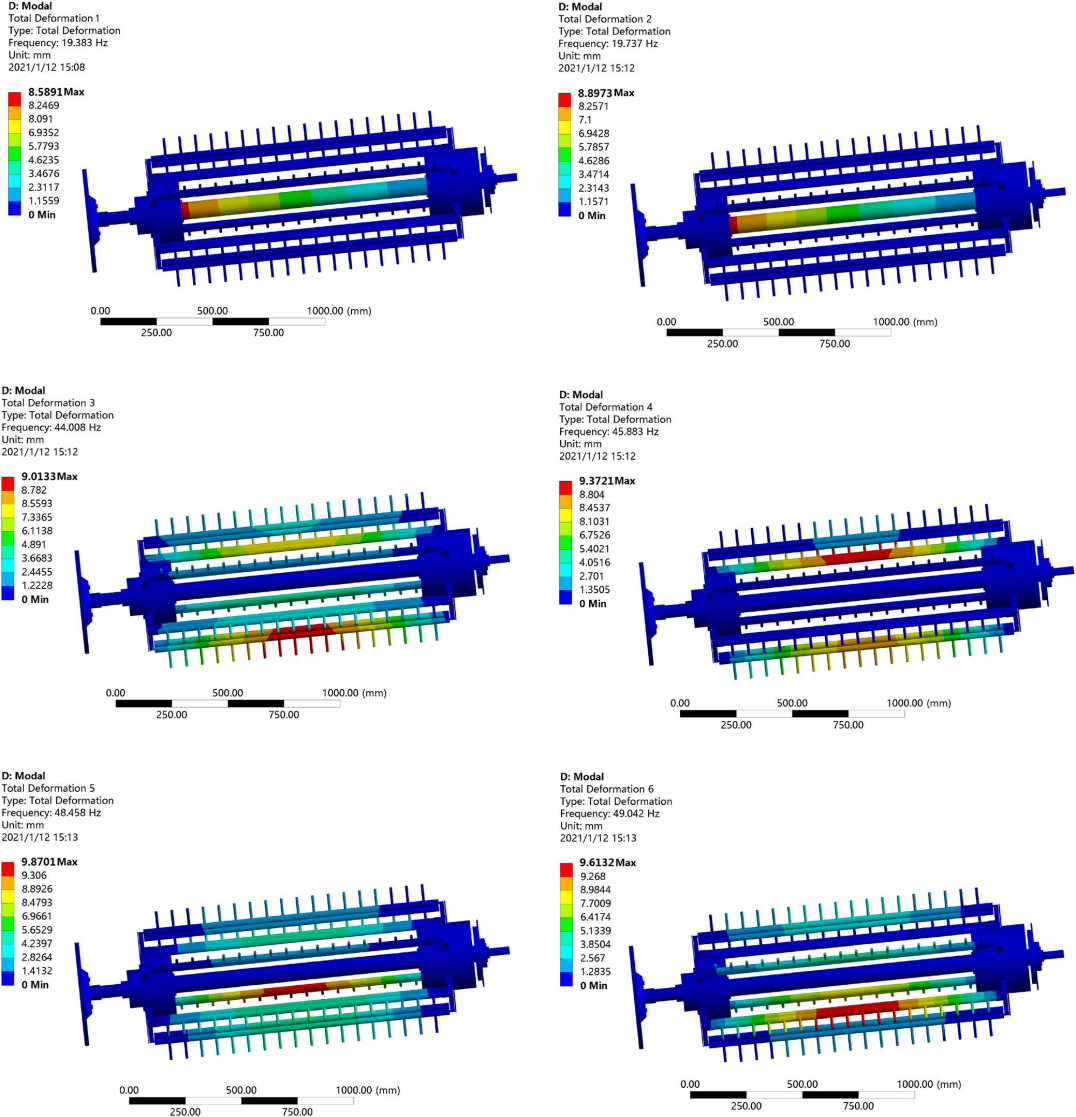
Six orders of natural frequency displacement cloud map of threshing drum

In ANSYS Workbench, set the first six frequencies are automatically solved. The first‐order and the second‐order frequencies are 19.383 and 19.737 Hz, respectively. The frequencies of third to sixth orders are highly close, with an increase of about 25 Hz compared with the first‐order frequency. The vibration‐type diagrams of the first six orders of the threshing drum are obtained by solving ANSYS Workbench, as shown in Figure 11.

The inherent frequency, maximum deformation, and the deformation sites of the threshing drum are available from the displacement nephogram of the threshing drum inherent frequencies of the first six orders. Based on the modal analysis of the first six orders for the threshing drum and its center shaft, the maximum deformation is 9.8701 mm which is less than the minimum threshing gap, therefore, no interference is expected to occur between the threshing drum and the concave grid.

### 3.2 Dynamic balance detection of drum

**FIGURE 12:**
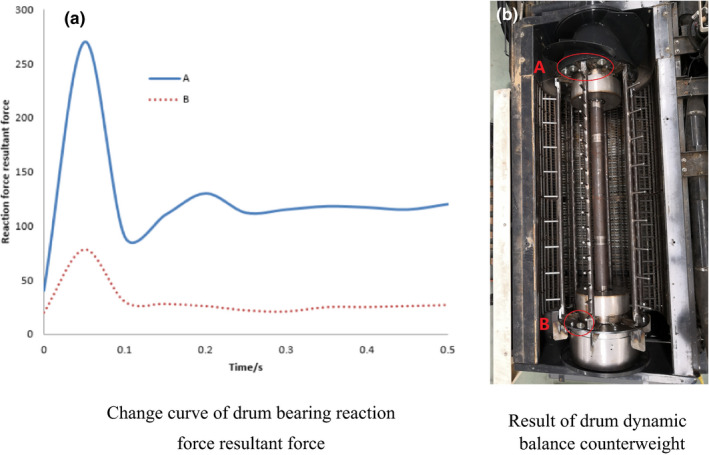
Test results of dynamic balance of drum, (a) Change curve of drum bearing reaction force resultant force, (b) Result of drum dynamic balance counterweight

Dynamic balance simulation results show that the values obtained as the resultant forces of the bearing reaction force at the end A and end B of the drum (end A is the feeding end and end B is the tail of the drum) are 162.1 N and 36.9 N, respectively, which after calculation, exceed the national standards ones. By loading the simulated counterweight at both ends of the drum, the dynamic balance simulation is carried out again, and the resultant forces of bearing reaction force at both ends of A and B are *F_A_*_ _= 108.9 N and *F_B_*_ _= 31 N, respectively. As shown in Figure 12a, the resultant force of bearing reaction force at both ends of A and B fluctuates greatly in the acceleration stage, and once the speed gets stable, the resultant force of bearing reaction force becomes the final calculation basis.

The residual unbalance value *U* must not be larger than the permissible residual unbalance value U_per_, that is, *U* < *U*_per_.

(10) $$U_{\mathrm{per}}=1000\frac{\left(e_{\mathrm{per}}\times \omega \right)m}{\omega }$$

where, *U* per is permissible residual unbalance, g/mm;( e per × ω) is balance quality level magnitude, mm/s; m is drum mass, kg; *ω* is drum angular velocity, rad/s. The corresponding balance quality grade value (e per × ω) of agricultural machinery is 16 mm/s, the mass of transverse axial flow drum is 150.5 kg, and ω is 62.8 rad/s. Then

(11) $$U_{\mathrm{per}}=1000\frac{\left(e_{\mathrm{per}}\times \omega \right)m}{\omega }=1000\times \frac{16\times 150.5}{62.8}=38344g/mm$$

the residual unbalance values at both ends of A and B

(12) U=F/ω

(13) $$U_{A}=\frac{108.9}{62.8^{2}}=27612g/mm$$

(14) $$U_{B}=\frac{31}{62.8^{2}}=7860g/mm$$

The total unbalance value is *U_A_* + *U_B_* = 35472 g/mm <*U_per_*, in compliance with the above‐mentioned requirements. Based on the numerical simulation and theoretical calculation of dynamic balance, YYW‐300 hard‐supported dynamic balance machine (Hoffmann) is used to check the dynamic balance of variable diameter threshing drum according to the national requirements of dynamic balance test standard (GB/T 9,239.1—2006 [ISO 1940–1–2003]). The counterweight iron blocks are installed inside the support plate at A and B ends, respectively. After re‐check, the counterweight finally meets the requirements of the national standard. The counterweight results are shown in Figure 12b.

### 3.3 Analysis of test results

**FIGURE 13:**
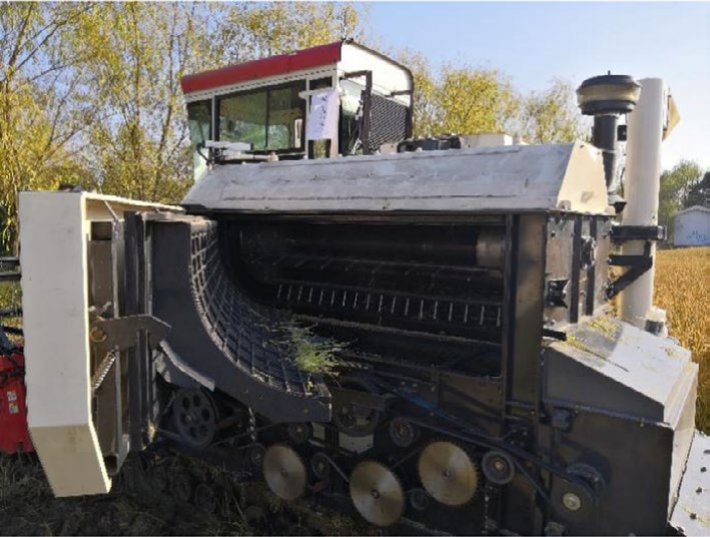
Field test

The assembled variable diameter threshing drum is installed on the combine harvester for the field test, as shown in Figure 13. Taking the drum rotation speed, the threshing gap, and the feeding rate as the experiment factors, the grain separation loss rate and the damage rate as performance indexes, the orthogonal rotation combination test was established (Table 3). The orthogonal rotation test results are obtained through field test, as shown in Table 4.

**TABLE 3:** Coding table of level of factor

Level	Feeding rate *X_1_* (kg.s−1)	Threshing gap *X_2_* (mm)	Drum speed *X_3_* (r/min)
+1.682	4	30	700
+1	3.6	26	660
0	3	20	600
−1	2.4	14	540
−1.682	2	10	500

**TABLE 4:** Quadratic regression orthogonal rotation test

N_O_.	*X_1_* (kg·s−1)	*X_2_* (mm)	*X_3_* (r/min)	*Y_1_* (%)	*Y_2_* (%)
1	−1	−1	−1	0.54	0.39
2	1	−1	−1	0.65	0.68
3	−1	1	−1	0.68	0.29
4	1	1	−1	0.84	0.55
5	−1	−1	1	0.31	0.91
6	1	−1	1	0.39	0.81
7	−1	1	1	0.31	0.57
8	1	1	1	0.82	0.86
9	−1.682	0	0	0.34	0.45
10	1.682	0	0	0.68	0.88
11	0	−1.682	0	0.69	0.76
12	0	1.682	0	0.77	0.27
13	0	0	−1.682	0.68	0.47
14	0	0	1.682	0.32	0.90
15	0	0	0	0.37	0.38
16	0	0	0	0.33	0.48
17	0	0	0	0.43	0.41
18	0	0	0	0.47	0.33
19	0	0	0	0.38	0.36
20	0	0	0	0.33	0.41
21	0	0	0	0.41	0.42
22	0	0	0	0.35	0.32
23	0	0	0	0.38	0.31

The obtained values for simplified regression equations of separation loss rate *Y_1_* and damage rate *Y_2_* were shown as follows:

(15) $$Y_{1}=0.38+0.1X_{1}+0.06X_{2}‐0.11X_{3}+0.06X_{1}X_{2}+0.04X_{1}^{2}+0.12X_{2}^{2}+0.036X_{3}^{2}$$

(16) $$Y_{2}=0.38+0.11X_{1}‐0.98X_{2}+0.14X_{3}+0.1X_{1}^{2}+0.047X_{2}^{2}+0.11X_{3}^{2}$$

The variance analysis to regression Equations (15) and (16) is shown in Table 5. The regression Equations (14) and (15) are very significant at the level when alpha = 0.05, and the values of lack of fit are *F_separation_*_ _= 3.40 and *F_damage_*_ _= 3.24, respectively, which are less than F 0.05 (5, 8) = 3.69, and show no significance, and the equation fits well. Therefore, the regression model can realize the prediction of test indexes and the control of parameters.

**TABLE 5:** Significance test results of regression equation

Index	Source of variance	D*_f_*	Ss	Ms	F	Significance
*Y_1_*	Regression analysis	9	0.68	0.075	17.98	Yes
	Residual	13	0.052	0.004		
	Lack of fit	5	0.037	0.007	3.40	No
	*X_1_*	1	0.15	0.15	35.92	Yes
	*X_2_*	1	0.059	0.059	14.02	Yes
	*X_3_*	1	0.16	0.16	38.66	Yes
*Y_2_*	Regression analysis	9	0.98	0.11	18.78	
	Residual	13	0.075	0.005		
	Lack of fit	5	0.05	0.01	3.24	No
	*X_1_*	1	0.16	0.16	27.15	Yes
	*X_2_*	1	0.13	0.13	22.91	Yes
	*X_3_*	1	0.28	0.28	48.88	Yes

### 3.4 Effect of experimental factors on separation loss rate

**FIGURE 14:**
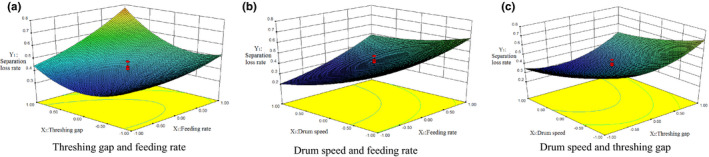
The effects of three experimental factors on separation loss rate, (a) Threshing gap and feeding rate, (b) Drum speed and feeding rate, (c) Drum speed and threshing gap

Design‐Expert software is used to draw the three‐dimensional factor response surface effect map. As shown in Figure 14, in the interaction between feeding rate *X_1_* and threshing gap *X_2_*, the separation loss rate *Y_1_* increases with the increase of feeding rate *X_1_*. With the increase in the value of *X_2_*, the value of *Y_1_* decreases first and then increases, and the value of *Y_1_* reaches its lowest level near the value of *X_2_* = 0. This is because as the value of *X_2_* increases, the grain thickness decreases, the grain is more likely to pass the grain layer, accompanied by a gradual decrease of *Y_1_*. However, a higher *X_2_* value is not conducive for grain separation, so the value of *Y_1_* will increase; the interaction between feeding rate *X_1_* and drum speed *X_3_* have a great impact on separation loss rate *Y_1_*. For example, the value of *Y_1_* increases when the value of *X_1_* increases, and decreases when the value of *X_3_* increases, *Y_1_* reaches its lowest value near the value of *X_1_* = −1 and the value of *X_3_* = 1, because as the value of *X_1_* increases, the thickness of grain layer in threshing device increases, and the threshing grain is less likely to pass the grain layer decreases, accompanied with the gradual increase of the value of *Y_1_*. Meanwhile, with the increase of the value of *X_3_*, the centrifugal force of material in the threshing device increases and the material layer thickness decreases, which is more conducive for grain separation. Therefore, the value of *Y_1_* will gradually decrease. In the interaction between *X_2_* and *X_3_*, the value of *Y_1_* will increase with the increase of the value of *X_1_* and decrease first and then increase with the increase of the value *X_2_*. *Y_1_* will reach its lowest value near the value *X_3_* = 1. It can be seen from the response surface in the figure that the drum speed *X_3_* has the greatest influence on the separation loss rate *Y_1_*, the threshing gap *X_2_* is second, and the feeding rate *X_1_* is the smallest.

It can be concluded that the separation loss rate increases with the increase in feeding rate, decreases first and then increases with the increase in threshing gap, and decreases with the increase in drum speed. This is in agreement with studies by Dainius et al. (2018) who have studied the loss rate of grain under different gaps and rotation speeds. They found that when the threshing gap is too small, the entrainment loss rate increase with the increase in the rotation speed of the drum. When the threshing gap was increased, the entrainment loss rate would decrease with the increase in the rotation speed of the drum. However, when the rotation of the drum was relatively low, the entrainment loss rate would increase gradually with the increase in threshing gap. When the rotation speed was increased, the entrainment loss rate would decrease gradually with the increase in threshing gap.

### 3.5 Effect of experimental factors on damage rate

**FIGURE 15:**
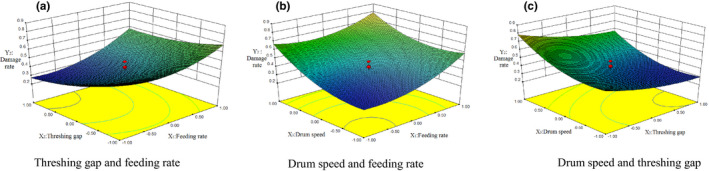
The effects of three experimental factors on damage rate, (a) Threshing gap and feeding rate, (b) Drum speed and feeding rate, (c) Drum speed and threshing gap

As seen from Figure 15, the interaction between feeding rate *X_1_* and threshing gap *X_2_*, the value of damage rate *Y_2_* increases with the increase of the value of *X_1_*. With the decrease of the value *X_2_*, the value of *Y_2_* increases gradually, and reaches its lowest level when near the value of *X_1_* = −1 and *X_2_* = 1, because with the decrease of the value of *X_2_*, the rubbing and grind action of grain in the grain layer becomes stronger, resulting in the gradual increase of the value of *Y_2_*; in the interaction between feeding rate *X_1_* and drum speed *X_3_*, the value of damage rate *Y_2_* increases with the increase of the values of *X_1_* and *X_3_*, and reaches its lowest level, near the value of *X_1_* = −1 and the value of *X_3_* = −1.2, because with the increase of the value of *X_1_*, the thickness of material layer in threshing device increases, more materials are added, and the actions such as rubbing, brushing and extrusion of grain exert increasing impact, resulting in the gradual increase of the value of *Y_2_*. At the same time, with the increase of the value of *X_3_*, the impact force and the number of hits on grain increases, resulting in the rapid increase of the value of *Y_2_*. In the interaction between threshing gap *X_2_* and drum speed *X_3_*, the value of damage rate *Y_2_* decreases with the increase of the value of *X_2_*, and increases with the increase of the value of *X_3_*, and reaches its lowest level near the value of *X_2_* = 1 and the value of *X_3_* = −1. It can be seen from the response surface in the figure that the drum speed *X_3_* has the greatest influence on the damage rate *Y_2_*, the threshing gap *X_2_* is second, and the feeding rate *X_1_* is the smallest.

It can be concluded that the damage rate increases with the increase in feeding rate, decreases with the increase in threshing gap, and increases with the increase in drum speed. This result is similar to the conclusion drawn from Xu et al. (2020) study, that is, with the increase in feeding rate, the damage rate will be higher. In the meantime, the damage rate will be higher with the increase in drum speed.

### 3.6 Performance index optimization

The separation loss rate and damage rate are the main indexes for evaluating the performance of threshing device, which should reach their minimum values under their respective constraints. According to the established mathematical models of separation loss rate *Y_1_* and damage rate *Y_2_*.

$$\left\{\begin{array}{cc} minY_{1}= & f\left(X_{1},X_{2},X_{3}\right)\\ minY_{2}= & f\left(X_{1},X_{2},X_{3}\right) \end{array}\right.$$

The constraint condition is.

$$\left\{\begin{array}{cc} y_{j}>0 & \left(j=1,2,3\right)\\ ‐1.682\leq x_{i}\leq 1.682 & \left(i=1,2,3\right) \end{array}\right.$$

**FIGURE 16:**
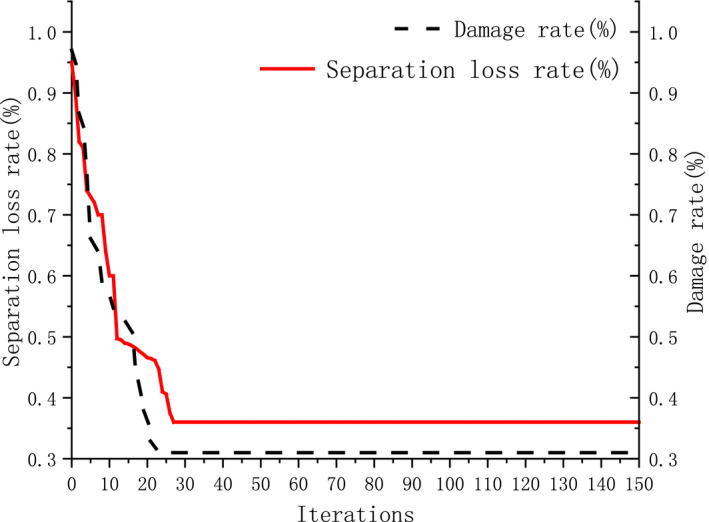
Iterative diagram of optimization function

The method of multiobjective optimization was used to analyze the threshing and separation and the best parameter combination is selected for comprehensive performance. By means of maximum modulus ideal point method, the weights selected for separation loss rate *Y_1_* and damage rate *Y_2_* are 6 and 4, respectively. By means of multi‐objective optimization, the optimum combination scheme of three factors was obtained as follows: The value of feeding rate is 2.2 kg/s, the value of threshing gap is 22 mm, the value of drum speed is 600 r/min, and the corresponding values of separation loss rate and damage rate are 0.35% and 0.31%, respectively. The iteration diagram of the optimization function is shown in Figure 16.

In order to verify the threshing performance of variable diameter drum, according to the test method described in Section 2.5, the test rice variety was still “Jia Hua,” Use the above optimum combination scheme to conduct field tests, the corresponding values of separation loss rate and damage rate 0.36% and 0.33%, respectively. The test results were similar to the calculation results (Xu, et al. 2020).

## 4 CONCLUSIONS

In this paper, according to the existing size of longitudinal axial flow drum on field test vehicle of combine harvester, the parameters of feeding section and threshing and separation section of variable diameter threshing drum are redesigned. On this basis, the diameter adjusting device and the adjusting self‐locking device were added to develop the variable diameter drum threshing device. The threshing performance of the variable‐diameter drum was tested through field experiments, and the following conclusions were obtained:

- The main design parameters of variable diameter threshing drum were determined. The length of the threshing and separation section of the threshing drum is 1,450 mm, the length of the feeding section is 350 mm, the reserved length of the self‐locking part is 300 mm. Force analysis was carried out on the device for diameter adjustment of variable diameter threshing drum, the design dimensions of the constant speed spiral disc and claws in the device for diameter adjustment were in compliance with the self‐locking conditions. The driving thread pitch of the threads is 8 mm, the spiral diameter is 150 mm. It can effectively ensure the reliability of the device for diameter adjustment, making it possible for the threshing drum to adjust the drum diameter integrally and quickly.
- Through the dynamic balance detection of the variable diameter drum, the total unbalance value is 35,472 g/mm, which in compliance with the requirements. Through the modal analysis of the threshing drum, the maximum deformation is 9.8701 mm, which is less than the minimum threshing gap, indicating that no interference is expected to occur between the threshing drum and the concave grid. The optimum working parameters combination was obtained through multi‐objective programming shown as follows: Feeding rate is 2.2 kg/s, threshing gap is 22 mm, and drum revolution speed is 600 r/min, the corresponding separation loss ratio and grain damage ratio were 0.35% and 0.31%, respectively. The field test results showed that the designed variable diameter threshing drum has a good threshing performance and stable operation efficiency. The drum diameter can be adjusted according to working conditions to improve the operation adaptability of the combine harvester.

## CONFLICT OF INTEREST

The authors declare that there is no conflict of interest.

## AUTHOR CONTRIBUTIONS

**Zhan Su:** Conceptualization (lead); Data curation (lead); Formal analysis (equal); Funding acquisition (equal); Investigation (equal); Methodology (equal); Resources (equal); Software (equal); Supervision (equal); Validation (equal); Writing‐original draft (equal). **Liquan Tian:** Data curation (equal); Funding acquisition (equal); Methodology (equal); Supervision (equal). **Zhao Ding:** Investigation (equal); Software (equal); Supervision (equal); Writing‐review & editing (equal). **Xue Lin:** Data curation (equal); Formal analysis (equal); Investigation (equal); Methodology (equal); Validation (equal); Visualization (equal). **Zhiming Wang:** Conceptualization (equal); Funding acquisition (equal); Software (equal); Supervision (equal); Writing‐review & editing (equal).
